# Common variation in *FAM155A* is associated with diverticulitis but not diverticulosis

**DOI:** 10.1038/s41598-020-58437-1

**Published:** 2020-02-03

**Authors:** Matthias C. Reichert, Juozas Kupcinskas, Antje Schulz, Christoph Schramm, Susanne N. Weber, Marcin Krawczyk, Christoph Jüngst, Markus Casper, Frank Grünhage, Beate Appenrodt, Vincent Zimmer, Algimantas Tamelis, Jaune I. Lukosiene, Neringa Pauziene, Gediminas Kiudelis, Laimas Jonaitis, Tobias Goeser, Maciej Malinowski, Matthias Glanemann, Limas Kupcinskas, Frank Lammert

**Affiliations:** 1 Department of Medicine II, Saarland University Medical Center, Saarland University, Homburg, Germany; 2 Department of Gastroenterology and Institute for Digestive Research, Lithuanian University of Health Sciences, Kaunas, Lithuania; 3 Department of General, Visceral, Vascular and Pediatric Surgery, Saarland University Medical Center, Homburg, Germany; 4 Clinic for Gastroenterology and Hepatology, University Hospital of Cologne, Cologne, Germany; 5 Laboratory of Metabolic Liver Diseases, Department of General, Transplant and Liver Surgery, Medical University of Warsaw, Warsaw, Poland; 6 Department of Gastroenterology and Hepatology, University Hospital Zurich, and University of Zurich, Zurich, Switzerland; 7 Department of Surgery, Lithuanian University of Health Sciences, Kaunas, Lithuania; 8 Institute of Anatomy, Lithuanian University of Health Sciences, Kaunas, Lithuania

**Keywords:** Genetic predisposition to disease, Colonic diseases

## Abstract

Colonic diverticulosis is a very common condition. Many patients develop diverticulitis or other complications of diverticular disease. Recent genome-wide association studies (GWAS) consistently identified three major genetic susceptibility factors for both conditions, but did not discriminate diverticulititis and diverticulosis in particular due the limitations of registry-based approaches. Here, we aimed to confirm the role of the identified variants for diverticulosis and diverticulitis, respectively, within a well-phenotyped cohort of patients who underwent colonoscopy. Risk variants rs4662344 in Rho GTPase-activating protein 15 (*ARHGAP15*), rs7609897 in collagen-like tail subunit of asymmetric acetylcholinesterase (*COLQ*) and rs67153654 in family with sequence similarity 155 A (*FAM155A*) were genotyped in 1,332 patients. Diverticulosis was assessed by colonoscopy, and diverticulitis by imaging, clinical symptoms and inflammatory markers. Risk of diverticulosis and diverticulitis was analyzed in regression models adjusted for cofactors. Overall, the variant in *FAM155A* was associated with diverticulitis, but not diverticulosis, when controlling for age, BMI, alcohol consumption, and smoking status (OR_adjusted_ 0.49 [95% CI 0.27–0.89], p = 0.002). Our results contribute to the assessment specific genetic variants identified in GWAS in the predisposition to the development of diverticulitis in patients with diverticulosis.

## Introduction

Colonic diverticulosis is a widespread gastrointestinal condition described as formation of diverticula, which are sac-like protrusions of mucosa and submucosa through muscularis externa^1,2^. Although diverticulosis is predominantly asymptomatic, many patients develop symptoms^3,4^. These manifestations are classified as uncomplicated and complicated diverticular disease (DD), an inflammation of the tissue (diverticulitis) or diverticular bleeding. The prevalence of the disease and its complications is constantly increasing due to the aging populations^5,6^. Therefore, the identification of underlying pathobiological mechanisms of the disease is relevant for improving everyday clinical practice. It is hypothesized that environmental factors together with structural alteration of the colonic wall and remodeling of the enteric nervous system are all predisposing components in the development of colonic diverticula^5^. In recent years, epidemiological twin data have suggested that genetic factors are another major cofactor in the pathogenesis of DD^7,8^. Up to date, there has been relatively little research effort to determine these genes and their sequence variants that are associated with the development of DD^9^. Recently an Icelandic study group published a genome-wide association study (GWAS) searching for sequence variants that affect the risk of developing DD in the Icelandic population, containing a replication cohort of Danish individuals with DD^10^. The initial analysis included 15,220 Icelanders who were tested for associations with DD (5,426 cases) and its more severe form diverticulitis (2,764 cases). Subsequently, after applying weighted thresholds^11^, 16 sequence variants identified in the GWAS were followed up in a DD sample from Denmark with 5,970 cases and 3,020 controls. In the combined analysis of these sample sets, three genetic loci that show genome wide-significance and may be associated with the risk of DD and/or diverticulitis were identified: intronic variants at the *ARHGAP15* (Rho GTPase-activating protein 15), *COLQ* (collagen-like tail subunit of asymmetric acetylcholinesterase) and *FAM155A* (family with sequence similarity 155 A) loci were significantly associated with DD. The second GWAS^12^ included 27,444 patients from the European component of the UK Biobank resource and compared them with 382,284 controls. Overall, 154 associated variants were further tested in 31,221 patients from the Michigan Genomics Initiative, finally confirming 42 associated variants including the three previously identified variants^10^. Most recently, a third european GWAS^13^ containing 451,099 patients in addition to identification of further loci, also confirmed these three major loci.

Notably, the dissection of the specific phenotypes diverticulosis and diverticulitis was incomplete in the GWAS, since the assessment of diverticulosis or diverticulitis is based on the ICD code. The studies applied the corresponding ICD codes in the ICD10 (K572-K579) and ICD9 (K562.10-13) systems, which also encompasses patients with diverticulitis (as well as diverticular bleeding), among the patients with diverticulosis. Even though ICD codes can identify patients with diverticulosis and DD, they do not discriminate between diverticulosis and diverticulitis^14^. A subanalysis of the patients from the Icelandic subcohort of the Icelandic/Danish GWAS included only patients with either surgically treated or complicated diverticulitis^10^, whereas mild and often also outpatient-treated cases of diverticulitis were probably mostly missed. This information is not available in the Danish subcohort at all. Additionally, patients with asymptomatic diverticulosis were not analyzed in these GWAS studies. A part of our samples were used in the GWAS from Schafmayer *et al*.^13^, and after adding further samples re-analyzed using additional clinical covariates from the database as outlined below and specifically focusing on patients with diverticulosis no prior diverticulitis as controls and (endpoint diverticulitis) and healthy with no diverticula (endpoint diverticulosis).

In this study, we therefore aimed to evaluate the associations between three major SNPs reported in the Icelandic GWAS applying weighted thresholds and confirmed in the North-American^12^ and European^11,13^ GWAS: *ARHGAP15* (rs4662344), *COLQ* (rs7609897) and *FAM155A* (rs67153654). The risk of developing diverticulosis and diverticulitis, respectively, was determined in a Caucasian (german/lithuanian) cohort phenotypically characterized for the specific phenotypes diverticulosis and diverticulitis. Due to the similar genetic background of Germans and Lithuanians^15^, a combined analysis was performed.

## Patients and Methods

### Study population

All patients taking part in the study were recruited at the Department of Medicine II, Saarland University Medical Center, Homburg, the Clinic for Gastroenterology and Hepatology, University Hospital of Cologne, Cologne, Germany, and the Department of Gastroenterology at the Lithuanian University of Health Sciences, Kaunas in Lithuania between 2012 and 2016 from patients referred for colonoscopy. A part of the samples from the Lithuanian cohorts came from our previous studies on colonic diseases and diverticulosis^16–18^ and were also used in a previous GWAS with less clinical information^13^. All patients and controls were of self-reported Caucasians ancestry (including grandparents). Risk factors, epidemiological and baseline data were assessed using a structured interview, performed by a physician assisting the patients with the questionnaires. The presence of diverticula was assessed by colonoscopy in all patients, which is the most widely accepted standard to detect diverticula. Only patients with complete colonoscopy including inspection of the cecum and at least adequate preparation, as assessed by the physician performing colonoscopy, were included in the study. All colonoscopies were performed using digital video endoscopes (high-resolution scopes Olympus CF 160, 180 or 190) by a senior gastroenterologist. Intestinal lavage for endoscopic examination was performed using 2 liters of a solution containing polyethylene glycol. Patients with inherited connective tissue disorders such as Ehlers-Danlos- or Marfan syndrome, non-Caucasian ethnicity or relatives of included patients were also excluded. The diagnosis of diverticulitis was established according to the current classifications for DD^19,20^. It was based on imaging by either computed tomography and/or ultrasound imaging as well corresponding clinical (pain in the lower left abdomen) and laboratory characteristics (increased serum inflammation markers). Suspected complicated diverticulitis was assessed with computed tomography in all cases. The study protocol was approved by the Research Ethics Committee of the Saarland University (approval 63/11), the Research Ethics Committee of the University of Cologne (approval 16–397) and the Regional Kaunas Ethics Committee (protocol No BE-10-2). The study was performed according to the Declaration of Helsinki. All patients have signed an informed consent form to participate in the study. For the purpose of this study, cases were defined as patients with diverticulosis or diverticulitis, respectively.

### DNA extraction and genotyping

Genomic DNA was extracted from peripheral blood mononuclear cells using the DNeasy Blood& Tissue Kit (Qiagen, Hilden, Germany). DNA concentrations were measured using a NanoDrop spectrophotometer. DNA samples were stored at −20 °C until analysis. Genotyping of the three genetic polymorphisms (rs4662344, rs7609897 and rs67153654) with Taqman assays was performed in 856 patients with diverticulosis and 479 controls of Caucasian descent in our accredited laboratory (DIN EN ISO 15189) in Homburg by a technician blinded to the phenotype of the patients. The fluorescence data was analyzed with allelic discrimination 7500 software v.2.0.6.

### Statistical analysis

Statistical Package for the Social Sciences (SPSS, Version 20, IBM, Munich, Germany) was used for statistical analysis. Power calculations were performed using PS (http://biostat.mc.vanderbilt.edu/wiki/Main/PowerSampeSize) to detect a significantly increased OR of 2 with a power of 80%, based on the corresponding frequencies of the risk alleles in rs4662344, rs7609897 and rs67153654, and assuming type I error rates of 0.05. Quantitative data were expressed as medians and ranges. Comparisons of frequencies of genotypes at the three loci were performed in 3 × 2 contingency tables listing cases and controls. Genotypic and allelic association tests were performed using χ^2^-square or Fisher’s exact tests (https://ihg.gsf.de/cgi-bin/hw/hwa1.pl). Due to the few homozygous mutants of the risk alleles we applied a dominant model. Genotype association analysis between SNPs and diverticulosis was performed using multiple logistic regression models adjusted for age, BMI, smoking status, and alcohol consumption, assuming log-additive effects. P-values < 0.05 were considered statistically significant. Results are expressed as odds ratios (OR) and 95% confidence intervals (CI). Pairwise linkage disequilibrium (r^2^) was calculated utilizing LDpair^21^ using a caucasian reference population (CEU) of the identified variants in all three GWAS^10,12,13^.

## Results

### Patient characteristics

In total, 1,332 patients (634 men, 47.6%) were included. Table 1 summarizes the baseline data of this study cohort. Frequency of diverticulosis in our cohort was similar to prior data^20^. The median age was 64 years (IQR 55–72). Characteristics of the subgroup of patients with diverticulitis are presented in Supplementary Table 1. The call-rate for the variations were >95% for all variants. The genotype frequencies (cut-off P > 0.05) were in Hardy-Weinberg equilibrium (HWE) in all controls for the variants in *ARHGAP15* and *FAM155A*. The HWE for the variant in *COLQ* deviated in controls (both in diverticulosis and diverticulitis-analyses p < 0.001), and was not included in further analysis. The minor allele frequencies (MAF) (Table 2) were similar to prior data^10,12,13^. In comparison of patients with diverticulosis and controls, patients with diverticulosis were significantly (P < 0.001) older and more obese (P < 0.001) than individuals with no diverticulosis. When comparing patients with diverticulitis, with diverticulosis and no prior diverticulitis, patients with diverticulitis where significantly younger (P < 0.001), more often smokers (P = 0.006), and more frequently current alcohol drinkers (P = 0.001). No association was detected for BMI (P = 0.20) and gender (P = 0.26). Table 3 presents the data on linkage disequilibrium (LD) of all variants identified in the GWAS^10,12,13^^.^

**Table 1:** Baseline data of study population. Values are given as median and interquartillic range (IQR), or frequencies and percentages. BMI = body mass index; Significant *P* values are highlighted in bold. *Diverticulosis versus healthy controls, **Diverticulitis versus Diverticulosis with no prior diverticulitis.

Parameter	Diverticulosis (n = 858)	Controls (n = 474)	Diverticulitis (n = 198)	Total (n = 1332)	P value*	P value**
Age, years	67 (59–74)	57 (46–66)	61 (53–71)	64 (55–72)	<**0.001**	**<0.001**
BMI, kg/m^2^	27.9 (25.2–31.6)	26.7 (23.7–30.3)	27.1 (24.8–30.5)	27.5 (24.8–31.2)	<**0.001**	0.20
Gender, men/women	424 (49.4%)	210 (44.3%)	105 (53.0%)	634 (47.6%)	0.08	0.26
Smoking ever, yes/no	300 (35.6%)	168 (36.7%)	86 (43.9%)	468 (36.0%)	0.72	**0.006**
Alcohol (daily), yes/no	50 (6.0%)	21 (4.6%)	19 (9.9%)	71 (5.5%)	0.31	**0.01**

**Table 2:** Genotypic and allelic frequencies in *ARHGAP15* and *FAM155A* of the combined German/Lithuanian cohort and published data from published GWAS.

Gene	A_min_/A_maj_	MAF (%)	CT (%)	TT (%)	OR	P_trend_	OR (95% CI)	P_allelic_	OR (95% CI)	P_genotypic_
***ARHGAP15*** (rs4662344)										
Diverticulitis*	T/C	19.5	32.8	3.1	1.20	0.16	1.27 (0.91–1.76)	0.16	1.38 (0.67–2.84)	0.38
Diverticulosis**	T/C	19.5	29.7	4.7	1.30	0.02	1.28 (1.00–1.63)	0.05	1.85 (0.97–3.50)	0.06
Controls_(no diverticulosis)_	T/C	15.9	26.3	2.7						
Controls_(no prior diverticulitis)_	T/C	18.8	28.8	4.4						
Iceland GWAS^10^_Diverticular disease***_	T/C	20.9							1.23 (1.17–1.29)	<0.001
Iceland GWAS^10^_Controls***_	T/C	17.7								
MGI GWAS^12^_****__Diverticular disease_	T/C	19.1/20.0							0.88/0.89	<0.001/0.07
MGI GWAS^12^_****__Controls_	T/C	17.3/18.0								
European GWAS^13^_Diverticular disease_	T/C	17.9******								<0.001
European GWAS^14^_Controls_	T/C	17.9******								
***FAM155A*** (rs67153654)			TA (%) AA (%)							
Diverticulitis*	A/T	14.1	21.9	3.1	0.68	0.01	0.66 (0.47–0.92)	0.01	0.45 (0.19–1.09)	0.07
Diverticulosis**	A/T	22.8	35.4	5.1	1.01	0.90	1.01 (0.81–1.27)	0.91	1.02 (0.61–1.72)	0.93
Controls (no diverticulosis)	A/T	22.6	35.2	5.1						
Controls (no prior diverticulitis)	A/T	24.3	37.1	5.8						
Iceland GWAS^10^_Diverticular disease***_	A/T	17.0							0.87 (0.83–0.91)	<0.001
Iceland GWAS^10^_Controls***_	A/T	18.6								
MGI GWAS^12^_*****__Diverticular disease_	A/T	17.0/17.8							0.87/0.90	<0.001/0.024
MGI GWAS^12^_*****__Controls_	A/T	19.0/19.5								
European GWAS^13^_Diverticular disease_	A/T	19.6******								
European GWAS^13^_Controls_	A/T	19.6******								

Genotypic and allelic frequencies of the variants. Values are given as count and percentage. A_maj_ = major allele; A_min_ = minor allele; CI = confidence interval; MAF minor allele frequency; MGI Michigan Genomics Initiative; OR = odds ratio. P Values calculated for *Diverticulitis versus Diverticulosis with no prior diverticulitis; **Diverticulosis versus healthy controls; ***refers to combined Icelandic/Danish sample; ****Data from variant rs6734367 which is in LD (r^2^ = 0.82) with variant rs4662344 in caucansians^31^, data from UK biobank/MGI; *****Data from the variant rs11619840 which is in LD (r^2^ = 0.93) with variant rs67153654 in caucansians^31^, data from UK biobank/MGI; ******Data only combined cases/controls available.

**Table 3:** Pairwise linkage disequilibrium (r^2^) calculated using LDpair (Machiela *et al*.^31^) with a caucasian reference population (CEU) of the identified variants in all three GWAS^10,12,13^. Bold values indicate where r^2^ is >0.8 representing strong LD. Variants from the initial GWAS^10^ are marked in underline.

		Variant present with significant association in GWAS (Reference number)	*ARHGAP15*		*FAM155A*				
			rs4662344	rs6734367	rs9520344	rs11619840	rs67153654	rs9555371	rs9520339
*ARHGAP15*	rs4662344	^10,13^		**0.82**	0.001	0.019	0.014	0.009	0.001
	rs6734367	^12,13^			0.001	0.035	0.028	0.020	0.001
*FAM155A*	rs9520344	^12,13^				0.0021	0.0008	0.0043	**1.0**
	rs11619840	^12,13^					**0.93**	**0.83**	0.0021
	rs67153654	^10,13^						**0.90**	0.0008
	rs9555371	^13^							0.0043
	rs9520339	^13^							

### Associations of variants and diverticulosis

Table 2 presents the allelic and genotypic frequencies comparing patients with diverticulosis to controls. MAF of the variant in *ARHGAP15* (rs4662344) was increased compared to controls, as described in the GWAS^10,12,13^. The major (T) allele of rs4662344 in *ARHGAP15* was significantly (OR 1.28; 95% CI 1.00–1.63) associated with diverticulosis. This association did not withstand after adjusting for corresponding environmental cofactors though (OR 1.22; 95% CI = 0.93–1.61) (Table 4). Neither was the MAF of the variant in rs67153654 in *FAM155A* different between cases and controls, nor could an association with diverticulosis be detected (Table 4).

**Table 4:** Multivariate analysis of factors associated with diverticulosis versus controls. CI = confidence interval.

Parameter	Adjusted OR* (95% CI)	P value
*ARHGAP15* rs4662344:T (cc + tc vs tt)	1.22 (0.93–1.61)	0.15
*ARHGAP15* rs4662344:TC (ct vs tt + cc)	1.14 (0.86–1.52)	0.35
*ARHGAP15* rs4662344:TT (tt vs tc + cc)	1.89 (0.84–4.29)	0.13
*FAM155A* rs67153654:T (ta + aa vs tt)	1.09 (0.84–1.00)	0.53
*FAM155A* rs67153654:AT (ta vs tt + aa)	1.02 (0.78–1.30	0.89
*FAM155A* rs67153654:TT (tt vs at + aa)	1.19 (0.67–2.10)	0.56

OR = odds ratio. *Adjusted for age, BMI, alcohol and smoking status.

### Associations of SNPs and diverticulitis

The minor allele of the variant rs4662344 in *ARHGAP15* was more frequent in diverticulitis cases in comparison to controls with diverticulosis and no prior diverticulitis, as similarly described previously in the GWAS^10,12,13^. The MAF of the major (A) allele of rs67153654 in *FAM155A* was markedly (OR 0.66; 95% CI 0.47–0.92) reduced in patients with prior diverticulitis compared to controls (Table 2) as also previously described^10,12,13^. These associations remained significant after adjusting for environmental cofactors (Table 5). The variant rs4662344 in *ARHGAP15* was borderline significantly (OR 1.43; 95% CI 1.00–2.03; P = 0.05) associated with diverticulitis after adjusting for the corresponding cofactors. Even though hampered by small sample size, similar results (n = 64) were obtained when analyzing patients with surgically treated diverticulitis (Supplementary Tables 2 and 3).

**Table 5:** Multivariate analysis of factors associated with diverticulitis in patients with diverticulosis.

Parameter	Adjusted OR* (95% CI)	P value
*ARHGAP15* rs4662344:T (cc + tc vs tt)	1.43 (1.00–2.03)	**0.05**
*ARHGAP15* rs4662344:TC (ct vs tt + cc)	1.35 (0.44–1.94)	0.11
*ARHGAP15* rs4662344:TT (tt vs tc + cc)	1.40 (0.61–3.21)	0.43
*FAM155A* rs67153654:T (ta + aa vs tt)	0.70 (0.49–0.99)	**0.04**
*FAM155A* rs67153654:AT (ta vs tt + aa)	0.73 (0.51–1.06)	0.10
*FAM155A* rs67153654:TT (tt vs at + aa)	0.68 (0.27–1.69)	0.41

CI = confidence interval, OR = odds ratio. *Adjusted for age, BMI, alcohol and smoking status. Significant P values are highlighted in bold.

## Discussion

The aim of our present study was to assess the role of genetic variations consistently identified in the three large recent GWAS^10,12,13^ for the specific risks for diverticulosis and diverticulitis, respectively. Our results are in line with previous data concerning the association of diverticulosis with age and BMI as risk factors (diverticulosis)^22–24^, as well as alcohol consumption^25,26^ and smoking status^27–29^ as risk factors for diverticulitis.

Our major finding is that the rs67153654 risk allele in *FAM155* is significantly associated with diverticulitis after adjusting for cofactors, but not with diverticulosis. The data on the risk variant rs4662344 in *ARHGAP15* was less consistent, it was borderline significantly associated with both diverticulosis and diverticulitis, but for confirmation additional larger studies are warranted. MAF of the analyzed SNPs was similar to previous data^10,12,13,30^. Even though the variants in *ARHGAP15* and *FAM155A* initially discovered in the Icelandic/Danish GWAS^10^ are at partially different genetic positions on the same genes compared to the variants identified in the following GWAS^12,13^, their LD indicates their common heritability. Therefore, an analysis using the SNPs initially identified in the Icelandic GWAS^10^, which applied weighted thresholds, is justified.

All of the analyzed SNPs are located in introns, supporting a molecular mechanism at the level of RNA-expression in the surrounding gene or LD to another, yet unidentified causal variant.

One of the major strengths of our study is the availability of clinical and endoscopic data and covariates, allowing the exact separation of patients with uncomplicated diverticulosis from patients developing diverticulitis. Patients treated for diverticulitis as outpatients were also included in our analysis. Our study adds new insights into the susceptibility for diverticulitis, specifically attributing the association with the risk variant in *FAM155A* (protective effect) to diverticulitis, but not diverticulosis. Our study has certain limitations though, that have to be acknowledged. Due to the retrospective design of the study, we could not investigate the outcomes of diverticulosis and diverticulitis, including DD-associated mortality. Even though a similar genetic background is shared between Germans and Lithuanians^15^ our results can not necessarily be transferred to non-Caucasians and have to be validated in other ethnicities ethnicity-specific analysis was not feasible due to sample size. Furthermore, several other variants associated to DD in the prior GWAS were not assessed in our analysis, and could also contribute significantly to the genetic risk to develop diverticulitis in patients with diverticulosis. Ultimately, the development of a genome-wide polygenic score^31^ should be strived for in both clincal entities diverticulosis and diverticulitis. The role of the risk variants in complicated DD could also not be explored, as our sample size for these additional subgroups was too small. Additionally, to the best of our knowledge only one study from 2010 assessing the application of ICD codes for the discrimination of diverticulosis and diverticulitis is available. Therefore, confirmatory investigations are necessary. Further studies are also needed to understand which of the variants in *FAM155A* are the causal mutations. Furthermore, as the function of the involved genes is largely known, further elucidation of the molecular background of *FAM155A* deficiency in the pathogenesis of diverticulitis is warranted.

## Conclusions

Our results indicate, that the variant in *FAM155A* is associated with diverticulitis, but not diverticulosis in Caucasians, whereas a risk variant in *ARHGAP15* might be associated with both diverticulosis and diverticulitis. Our results contribute to the assessment of these genetic variants identified in GWAS in the predisposition to the development of diverticulitis in patients with diverticulosis.

## Supplementary information

Supplementary Data 1.

## Data Availability

The datasets generated during and/or analyzed during the current study are available on request as permitted by data protection laws and patients consent.
