# Supplementary material for: Common variation in FAM155A is associated with diverticulitis but not diverticulosis
Source: Sci Rep. 2020 Feb 3;10:1658. doi: 10.1038/s41598-020-58437-1 (PMC6997170; doi:10.1038/s41598-020-58437-1)
Supplement: Supplementary file 1 — Supplementary Data 1. [file 41598_2020_58437_MOESM1_ESM.docx]

**Common variation in *FAM155A* is associated with diverticulitis but not diverticulosis**

Matthias C Reichert^1*¶^, Juozas Kupcinskas^2*^, Antje Schulz^3^, Christoph Schramm^4^, Susanne N Weber^1^, Marcin Krawczyk^1,5^, Christoph Jüngst^1,6^, Markus Casper^1^, Frank Grünhage^1^, Beate Appenrodt^1^, Vincent Zimmer^1^, Aligamantas Tamelis^7^, Jaune I Lukosiene^2^, Neringa Pauziene^8^, Gediminas Kiudelis^2^, Laimas Jonaitis^2^, Tobias Goeser^4^, Maciej Malinowski^3^, Matthias Glanemann^3^, Limas Kupcinskas^2^ and Frank Lammert^1^

^*^These authors contributed equally to this work.

^1^Department of Medicine II, Saarland University Medical Center, Saarland University, Homburg, Germany

^2^Department of Gastroenterology and Institute for Digestive Research, Lithuanian University of Health Sciences, Kaunas, Lithuania

^3^Department of General, Visceral, Vascular and Pediatric Surgery; Saarland University Medical Center; Homburg, Germany

^4^Clinic for Gastroenterology and Hepatology, University Hospital of Cologne, Cologne, Germany

^5^Laboratory of Metabolic Liver Diseases, Department of General, Transplant and Liver Surgery, Medical University of Warsaw, Warsaw, Poland

^6^Department of Gastroenterology and Hepatology, University Hospital Zurich, and University of Zurich, Zurich, Switzerland.

^7^Department of Surgery, Lithuanian University of Health Sciences, Kaunas, Lithuania

^8^Institute of Anatomy, Lithuanian University of Health Sciences, Kaunas, Lithuania

Parameter Diverticulitis (n=197)

Surgically treated 64 (32.5)

Number of flares 1 (1-10)

Age at first flare (years) 58 (49-66)

≥3 Flares 42 (21.3)

**Supplementary Table 1.** Baseline data of diverticulitis patients. Values are given as

median and range (number of flares) interquartile range (age at first flare), or frequencies

and percentages.

Gene A_min_/A_maj_ MAF (%) fCT (%) fTT (%) OR P_trend_ OR (95% CI) P_allelic_ OR (95%CI) P_genotypic_

*ARHGAP15* **(**rs4662344)

Cases T/C 19.5 32.8 3.1 1.002 0.84 1.13 (0.66-1.93) 0.66 0.74 (0.17-3.21) 0.68

Controls T/C 18.8 28.8 4.4

*FAM155A* (rs67153654) fTA (%) fAA (%)

Cases A/T 14.1 21.9 3.1 0.55 **0.01** 0.44 (0.25-0.80) **0.01** 0.41 (0.10-1.76) 0.22

Controls A/T 24.3 37.1 5.8

**Supplementary Table 2.** Genotypic and allelic frequencies in *ARHGAP15* and *FAM155A* of 856 patients with diverticulosis with and without prior surgical diverticulitis.

Genotypic and allelic frequencies of the variants. Values are given as frequencies and percentage. Significant P values are highlighted in bold. Amaj = major allele;

Amin = minor allele; CI = confidence interval; MAF minor allel frequency OR = odds ratio.

Parameter Adjusted OR* (95% CI) P value

*ARHGAP15* rs4662344:T (CC+TC vs TT) 1.16 (0.66-2.02) 0.61

*ARHGAP15* rs4662344:TC (CT vs TT + CC) 1.26 (0.71-2.24) 0.42

*ARHGAP15* rs4662344:TT (TT vs TC + CC) 0.80 (0.18-3.60) 0.77

*FAM155A* rs67153654:T (TA + AA vs TT) 0.49 (0.27-0.89) **0.02**

*FAM155A* rs67153654:AT (TA vs TT + AA) 0.48 (0.26-0.91) **0.02**

*FAM155A* rs67153654:TT (TT vs AT +AA) 0.82 (0.19-3.60) 0.80

**Supplementary Table 3.** Multivariate analysis of factors associated with surgical diverticulitis in patients with diverticulosis

CI = confidence interval, OR = odds ratio.*adjusted for age, BMI, alcohol and smoking. Significant P values are highlighted in bold.
